# Mitotic degradation of yeast Fkh1 by the Anaphase Promoting Complex is required for normal longevity, genomic stability and stress resistance

**DOI:** 10.18632/aging.100949

**Published:** 2016-04-19

**Authors:** Mackenzie E. Malo, Spike D.L. Postnikoff, Terra G. Arnason, Troy A.A. Harkness

**Affiliations:** ^1^ Department of Anatomy and Cell Biology, University of Saskatchewan, Saskatchewan S7N 5E5, Canada; ^2^ Department of Medicine, University of Saskatchewan, Saskatchewan S7N 5E5, Canada

**Keywords:** Fkh1, Anaphase Promoting Complex (APC), yeast, ubiquitin signaling, mitosis, genetics

## Abstract

The *Saccharomyces cerevisiae* Forkhead Box (Fox) orthologs, Forkheads (Fkh) 1 and 2, are conserved transcription factors required for stress response, cell cycle progression and longevity. These yeast proteins play a key role in mitotic progression through activation of the ubiquitin E3 ligase Anaphase Promoting Complex (APC) via transcriptional control. Here, we used genetic and molecular analyses to demonstrate that the APC E3 activity is necessary for mitotic Fkh1 protein degradation and subsequent cell cycle progression. We report that Fkh1 protein degradation occurs specifically during mitosis, requires APC^Cdc20^ and proteasome activity, and that a stable Fkh1 mutant reduces normal chronological lifespan, increases genomic instability, and increases sensitivity to stress. Our data supports a model whereby cell cycle progression through mitosis and G1 requires the targeted degradation of Fkh1 by the APC. This is significant to many fields as these results impact our understanding of the mechanisms underpinning the control of aging and cancer.

## INTRODUCTION

The molecular genetics governing eukaryotic longevity are strongly conserved from yeast to mammals [1]. The genetic malleability of yeast has facilitated discoveries that have determined factors involved in both replicative (divisions of a single cell, mitotic) and chronological (population survival time, post-mitotic) lifespan, with corresponding orthologs in higher eukaryotes subsequently proven to carry out similar roles. Specifically, the technique of yeast replicative lifespan (RLS) follows how many mitotic divisions a yeast cell will go through prior to senescence, whereas chronological lifespan (CLS) measures how long a post-mitotic population of cells will remain metabolically active [2, 3]. These are two very different measures of the yeast life cycle, and are dictated by similar, yet distinct, sets of genes [3]. Intertwined with the genes affecting aging are those related to stress response and adaptive survival. The Anaphase Promoting Complex (APC) and the yeast Forkhead (Fkh) proteins (Fkh1 and Fkh2) interact to play important roles in both lifespan and stress response [4-6].

Stress response genes play a significant role in lifespan in multiple model systems [7-9]. The evolutionarily conserved Fox family of proteins are stress response transcription factors that are critical for properly controlling apoptosis, autophagy, metabolism and cell proliferation [10-12]. This, in turn, improves cell health, survival, and extension of lifespan. In higher eukaryotes including flies, mice, and worms, mutations of various Fox family members results in significant elevations of cancer incidence, supporting their role in apoptosis and proliferation [13, 14]. Higher eukaryotic Fox proteins undergo numerous post-translational modifications including phosphorylation, and ubiquitin-dependent degradation by the proteasome, leading to a variety of molecular outcomes [15, 16]. For example, phosphorylation of FoxO proteins by different upstream kinases, including AKT [15] or AMPK [17], affects FoxO nuclear localization and gene target expression. Here, we demonstrate that the yeast Fox ortholog, Fkh1, is also regulated at the level of protein degradation, highlighting that the Fkh system in yeast is a viable lower eukaryotic model for genetic and molecular investigations into lifespan and stress response mechanisms that will benefit our understanding of human lifespan and disease.

Yeast encode four Fox orthologues: Fkh1, Fkh2, Hcm1 and Fhl1 [18]. The Fkh1 and Fkh2 transcription factors are evolutionarily conserved, and at least Fkh1 and Fkh2 are redundant [5, 10, 19-21]. The Fkhs interact with the transcriptional cofactor Ndd1 in G2 to drive transcription of G2/M genes. In G1, Sir2 is recruited to promoters via the Fkh proteins to effect repression of the same key cell cycle progression genes activated earlier during G2 [19, 22, 23]. Because of their redundancy, genomic deletion of both *FKH1* and *FKH2* genes is required to assess their inherent function. The *fkh1Δ fkh2Δ* strain is exquisitely sensitive to oxidative stress and has a significantly shortened lifespan [5]. Conversely, elevated expression of either *FKH1* or *FKH2* independently enhanced lifespan, and augmented oxidative stress resistance [5].

An interdependence also exists in yeast between the APC and the Fkh proteins that impacts lifespan and stress response [5, 11]. Fkh1 and Fkh2 proteins can both activate the APC under normal growth conditions to coordinate cell cycle progression [5]. The APC is a multi-subunit ubiquitin ligase, or E3, that is predominantly known as being required for cell cycle progression through mitosis and for G1 maintenance, in lower and higher eukaryotes [24, 25]. Cdc20 controls APC function through mitosis, while Cdh1 regulates APC-dependent processes through G1 passage. We have described biological roles affected by the APC that go beyond lifespan, including critical functions in stress response, mitotic chromatin assembly, and mitotic-associated histone modifications [4, 26-29]. We observed that deletion of both *FKH* genes was necessary to further impair mutant APC phenotypes, such as sensitivity to temperature and oxidative stress, and reduced lifespan, indicating how important this combination of genes is to cell health and adaptive survival.

Activators of the APC, such as Clb2 and Cdc20, are often targeted for ubiquitin-dependent degradation through the E3 activity of the APC itself [30, 31]. Although we had evidence that the Fkh proteins likely activated the APC [5], we did not know if Fkh1 was targeted for degradation like other APC activators. Our hypothesis that Fkh1 served as an APC target grew from our observation that deletion of *FKH1* suppressed mutant APC defects. This is based on observations that deletion of APC targets, which accumulate in APC mutants, is predicted to alleviate APC mutant phenotypes [32]. Thus we queried if Fkh1 is also degraded in a cell cycle-dependent manner. We demonstrate here that the regulation of Fkh1 occurs at the onset of mitosis via targeted degradation initiated by the APC^Cdc20^ complex. Mutation of a highly conserved lysine stabilized Fkh1, conferred cell cycle, heat stress, and lifespan defects, but did not impair Fkh1/Apc5 interactions nor recruitment to promoters. These findings of conserved regulation of the Fox family of proteins from yeast to humans demonstrates that yeast provide valuable insight into conserved Fox molecular regulation mechanisms.

## RESULTS

### Deletion of *FKH1* suppresses APC mutant defects

**Figure 1:**
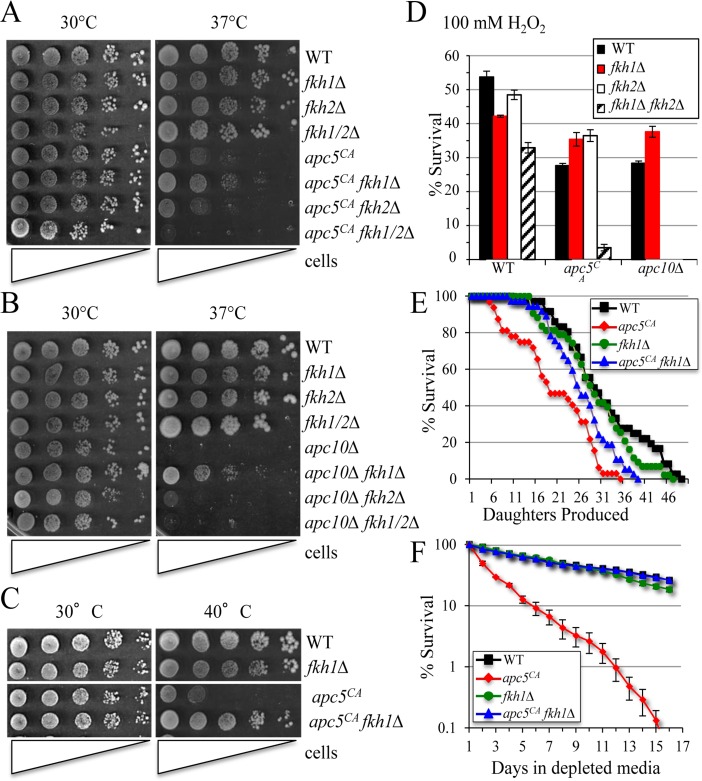
Deletion of *FKH1* reverses APC mutant phenotypes (**A**) The various yeast strains shown were grown overnight at 30°C. The next morning a 10-fold serial dilution series starting with 1 × 10^7^ cells/ml was spotted onto YPD plates and grown at 30°C and 37°C for 3 to 5 days. (**B**) The strains shown were treated as above. (**C**) Cells were spot diluted and grown at 30°C and 40°C to accentuate temperature sensitive growth. (**D**) The cells shown were grown to day 5 of stationary phase then split, with one half treated with 100 mM H_2_O_2_ for 1 hour. Equal numbers of cells were then plated onto YDP with all treated cell counts compared to untreated controls. The experiment was done in triplicate with standard error shown. (**E**) Replicative lifespan was performed with the cells shown. (**F**) Chronological lifespan of the cells used in (**E**) was performed in triplicate. Cells were grown in Complete Media (CM) and maintained in the Depleted Media (DM) once stationary phase was reached. Standard error of each timepoint is shown.

We have extensively used the *apc5^CA^* (chromatin assembly) mutant allele for the bulk of our genetic studies to gain insight into APC function [4-6, 26-29, 33]. We discovered the allele in a screen for chromatin assembly mutants [26]. This allele harbors a 2 bp deletion (_37_AT_38_), conferring a temperature sensitive phenotype (*ts*); growth is impaired at 37°C, versus the optimal growth temperature of 30°C. This allele is predicted to encode a short N-terminal portion of Apc5 (the first 12 Apc5 residues followed by 18 novel residues). However, since *APC5* is an essential gene and a short N-terminal portion of Apc5 does not rescue the *apc5^CA^ ts* defect [26, 34, 35], we fused the TAP epitope to the C-terminus of the *apc5^CA^* allele and discovered that the *ts* phenotype is due to an N-terminally truncated protein that likely starts from an internal methionine, and/or undergoes programmed ribosome frameshifting [36] (Fig. S1A). We previously used this allele to show that deletion of both *FKH1* and *FKH2* was necessary to further impair *apc5^CA^* mutant phenotypes [5]. However, here we show that deletion of only *FKH1*, and not *FKH2*, improved APC mutant phenotypes (Fig. 1) implicating Fkh1, but not Fkh2, as a potential APC target. This is consistent with other literature noting that deletion of either *FKH1* or *FKH2* alone has mild, but opposing and independent phenotypes [37, 38].

We began by examining APC mutant phenotypes, including temperature and oxidative stress sensitivity, and reduced lifespan. Deletion of only *FKH1* suppressed *ts* growth in both *apc5^CA^* and in the more severe *apc10Δ* mutant (Figs. 1A-C, S1B). The ability to improve *apc5^CA^ ts* growth was more apparent when the cells were grown at 40°C. Sensitivity of *apc5^CA^* cells to oxidative stress was also partially alleviated by deletion of *FKH1* or *FKH2*. In contrast, loss of *FKH1* in *apc10 Δ* cells suppressed oxidative stress phenotypes, while deletion of *FKH2* was lethal (Fig. 1D). These observations supported our consideration that Fkh1 may serve as an APC target. The remainder of this manuscript will focus on Fkh1-APC interactions in order to understand the molecular mechanisms regulating this interaction.

We had previously observed that overexpression of *FKH* genes was toxic to cells and induced apoptotic-like cell death [5]. Thus, it is likely that if Fkh1 is accumulating in *apc5^CA^* mutant cells, it may likewise be linked to a shorter lifespan. Our results confirm that deletion of *FKH1* completely restored *apc5^CA^* RLS (Fig. 1E) and CLS (Fig. 1F) to wild type (WT) levels. Thus, by removing Fkh1, stress and lifespan defects are reduced. Taken together, these observations suggest that Fkh1 plays a detrimental role in cells experiencing compromised APC function.

### Fkh1 accumulates in *apc5^CA^* cells in a ubiquitin- and proteasome-dependent manner

**Figure 2:**
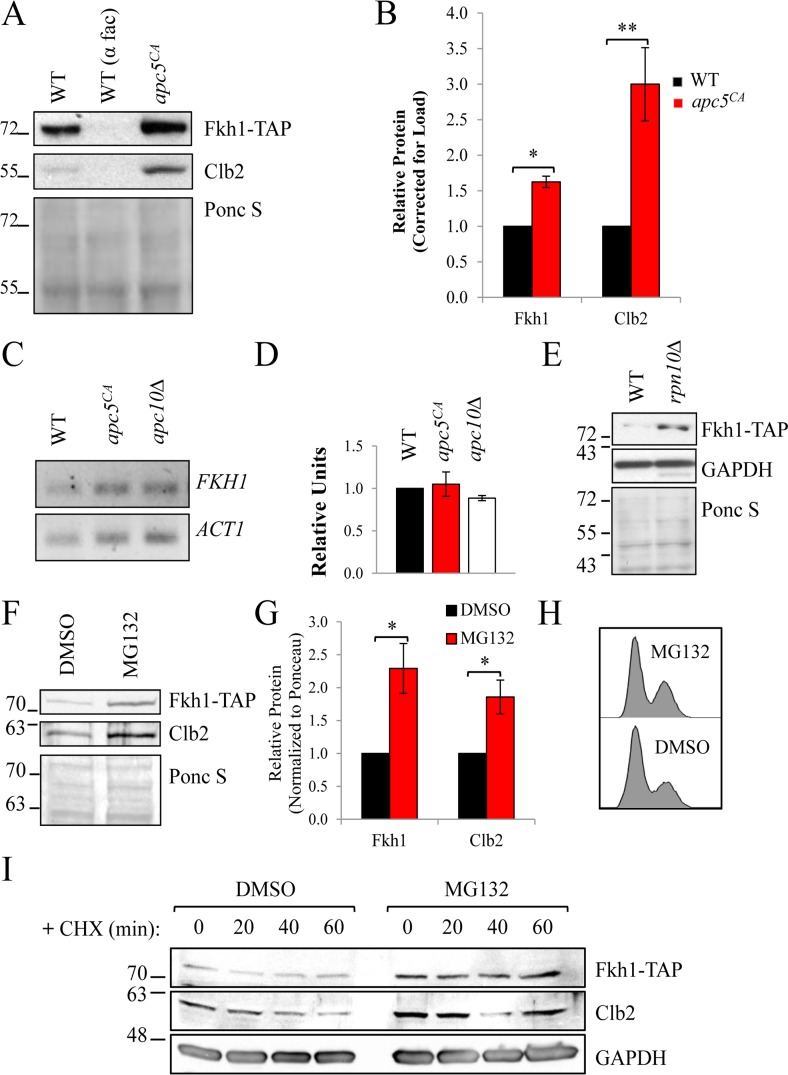
Fkh1 stability depends on the APC and the proteasome (**A**) WT and *apc5^CA^* cells expressing Fkh1-TAP were grown to mid log phase. WT cells were also treated with α-factor to arrest cells in G1. Whole cell lysates were prepared and analyzed by Western analyses with the antibodies shown. (**B**) The experiment in (A) was repeated 3 times, with all protein bands quantified, normalized to Ponceau S stained gels and plotted. Standard error is shown. * - p≤0.001; ** - p≤0.005. (**C**) The cells shown were grown to mid log phase, after which total RNA was extracted and used for quantitative reverse transcriptase PCR using primers against *FKH1* and *ACT1* as a normalization control. (**D**) The experiment in (C) was repeated 3 times with bands quantified, normalized to *ACT1*, and plotted. Standard error is shown. (**E**) WT and *rpn10Δ* cells expressing endogenous *FKH1-TAP* were grown to mid log phase, with proteins analyzed by Westerns using antibodies against TAP and GAPDH, with the Ponceau S stained gel confirming equivalency of load. (**F**) Asynchronous *FHK1-TAP* cells were incubated with 75 μM MG132 for 3 hours at 30°C prior to preparing cell lysates to inhibit the proteasome. Westerns were then performed using antibodies against TAP and Clb2. (**G**) The bands from (**F**) were quantified, normalized to the Ponceau S stained gel and plotted. * - p≤0.05. (**H**) Cells in (**F**) were used for flow cytometry to determine cell cycle distribution. (**I**) Asynchronous *FKH1-TAP* expressing cells, were treated +/− MG132 as in (**F**). Cycloheximide (CHX) was then added to inhibit protein synthesis with samples taken every 20 minutes to determine Fkh1-TAP stability.

To test the hypothesis that Fkh1 is targeted for ubiquitin-dependent degradation by the APC, we first assessed Fkh1 protein levels in asynchronous WT and *apc5^CA^* cells. We integrated Fkh1-TAP into the WT and *apc5^CA^* strains, expressed under its endogenous promoter. As a control, we followed the protein levels of a known APC targeted protein, Clb2, which is selected for degradation at the end of mitosis and is at minimal abundance in G1 [39]. Like Clb2, Fkh1 was dramatically reduced in G1 arrested cells and accumulated in *apc5^CA^* cells (Figs. 2A, 2B). The accumulation of Fkh1 protein in the *apc5^CA^* strain is most likely a post-translational event, as accumulation of Fkh1 protein did not correlate with increased mRNA transcript (Figs. 2C, 2D).

To determine whether Fkh1 G1 instability was dependent on the proteasome, we expressed endogenous Fkh1-TAP in *rpn10Δ* cells, which lack the proteasome ubiquitin receptor [40]. In *rpn10Δ* cells, potentially ubiquitinated proteins accumulate [6, 29], and this was observed for Fkh1-TAP (Fig. 2E). WT Fkh1-TAP expressing cells were next exposed to 75 μM MG132, a proteasome inhibitor, for 3 hours prior to harvesting proteins. In the presence of MG132, both Clb2 and Fkh1-TAP accumulated (Figs. 2F, 2G). This was not cell cycle related, as cells treated with MG312 had the same flow cytometry profile as untreated cells (Fig. 2H). Next, we added cycloheximide (CHX), which halts all protein synthesis, to MG132 treated cells to determine whether MG132 impacts Fkh1 stability or steady state levels. Fkh1-TAP and Clb2 protein levels were both elevated in MG132 treated cells, consistent with stable proteins (Fig. 2I).

**Figure 3:**
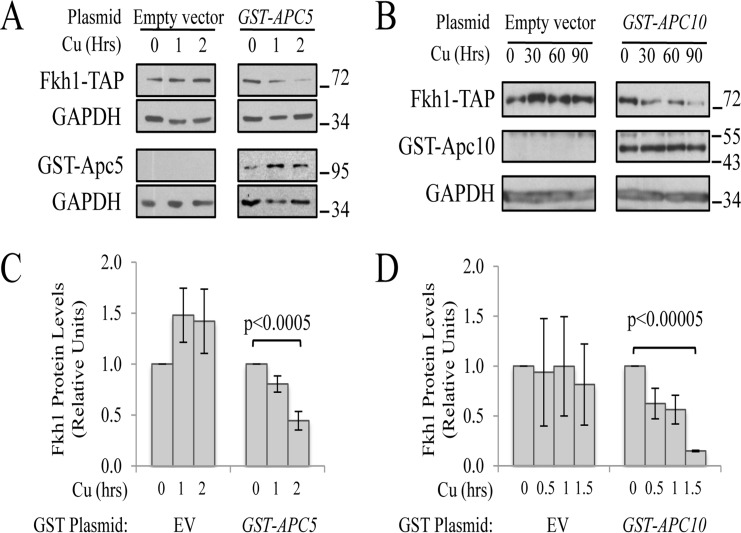
APC subunit content controls Fkh1 protein levels (**A**) Fkh1-TAP cells expressing plasmid-borne *GST-APC5*, or the empty vector control, were grown to early log and then exposed to 50 μM CuSO_4_ for 0, 1 or 2 hours. Cell lysates were prepared and analyzed using Westerns with antibodies against TAP, GST and GAPDH as a load control. (**B**) The same experiment as in (**A**) was performed except the plasmid expressed *GST-APC10*. (**C**) The bands from 3 repeat experiments in (**A**) were scanned, quantified, normalized to GAPDH, and plotted. (**D**) The bands from 2 repeat experiments in (**B**) were scanned and treated as in (**C**). Standard error of the mean is shown. Significance was calculated using a Student t-test.

If Fkh1 degradation is indeed dependent on the APC E3 activity, then increased APC subunit expression may further decrease Fkh1-TAP levels through enhanced E3 activity. When plasmids expressing copper-inducible promoter (*CUP1_prom_*) driven *GST*-*APC5* (Fig 3A) or *GST-APC10* (Fig. 3B) were expressed in Fkh1-TAP cells, we indeed observed that Fkh1-TAP levels decreased as compared to empty vector controls. Interestingly, Fkh1-TAP levels increased in response to copper alone, potentially due to induction of Fkh1 by the heavy metal stress. Taken together, these observations are consistent with the idea that Fkh1-TAP instability is APC, ubiquitin- and proteasome-dependent.

### Fkh1 is specifically targeted for degradation at the onset of mitosis by APC^Cdc20^

**Figure 4:**
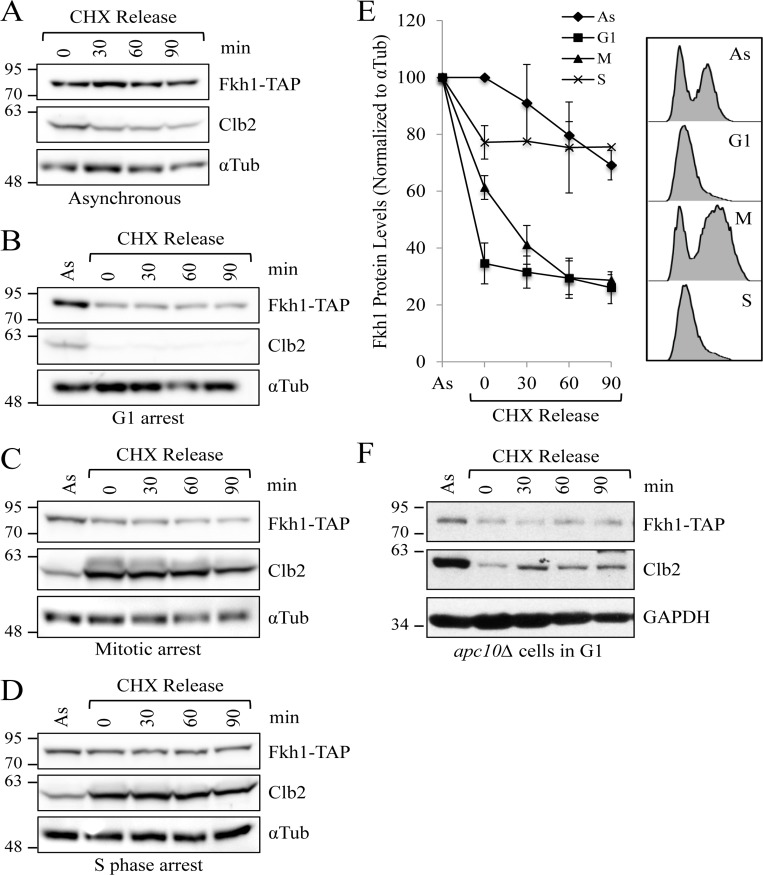
Fkh1 instability occurs specifically in mitosis (**A**) WT cells expressing endogenous *FKH1-TAP* were grown to mid log. CHX was added to the asynchronous cells to stop further protein synthesis. Samples were removed every 30 minutes and assessed by Western analyses with the antibodies shown. (**B**) The experiment described in (**A**) was performed, except the cells were arrested in G1 using α-factor. (**C**) The experiment described in (**A**) was performed, except the cells were arrested in mitosis using nocodazole. (**D**) The experiment described in (**A**) was performed, except the cells were arrested in S phase using hydroxyurea. (E) Blots from 3 repeat experiments of (**A**), (**B**), and (**C**), and 2 repeats of (**D**), were imaged with Fkh1-TAP levels compared over time to visualize Fkh1 stability. Standard error of the mean is shown. Samples were also removed following cell treatment for flow cytometry to verify cell cycle arrest. (**F**) *apc10Δ* Fkh1-TAP cells were arrested in G1 using α-factor. CHX was then added with samples removed every 30 minutes for protein analyses.

To determine whether Fkh1 stability is correlated with specific positions within the cell cycle, we arrested Fkh1-TAP expressing cells at various points in the cell cycle. CHX was then added to block further protein synthesis with samples removed every 30 minutes to assess protein stability from the arrest point forward (Figs. 4A-D). We observed that Fkh1-TAP was at its lowest level during G1, but remained stable (Figs. 4B, 4E). In contrast, Fkh1 is unstable during mitosis in the WT strain (Figs 4C, 4E). Stable Fkh1-TAP appears to again accumulate during S phase (Figs. 4D, 4E), consistent with cyclical *FKH1* mRNA expression throughout the cell cycle, which peaks during S phase [41]. In *apc10*Δ cells, Fkh1-TAP protein levels in G1 arrested cells, compared to G1 arrested WT cells, was higher when compared to asynchronous cells (Figs. 4F, S4C). Thus, these results indicate that Fkh1-TAP is specifically targeted for degradation at the onset of mitosis by the APC, which is also active at the onset of mitosis.

**Figure 5:**
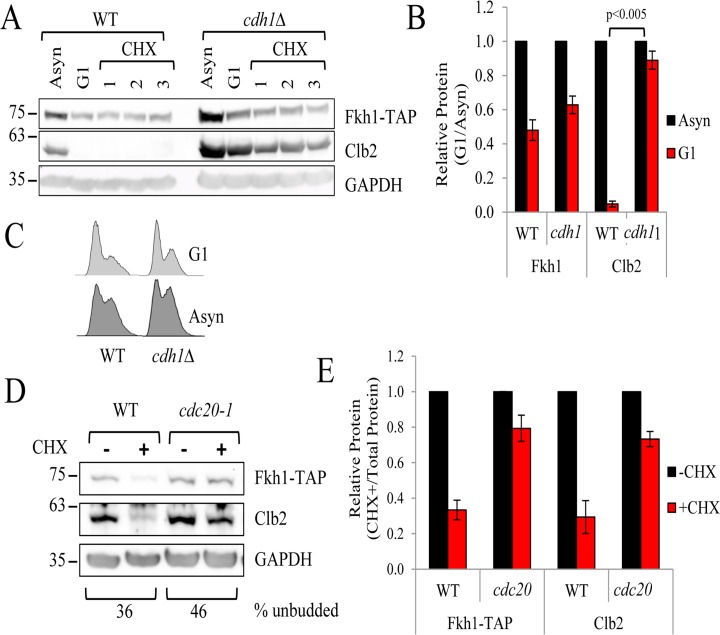
Fkh1 instability is determined by the APC^Cdc20^ complex (**A**) WT and *cdh1Δ FKH1-TAP* cells were arrested in G1 using α-factor for 3 hours, followed by the addition of CHX. Proteins were removed every hour to determine Fkh1-TAP stability. Clb2, a known target of the APC^Cdc20^ complex was also monitored as a control. (**B**) The experiment in (**A**) was repeated 3 times, with bands quantified, normalized to asynchronous controls, and plotted. Standard error and p-values are shown. (**C**) Asynchronous and G1 arrested WT and *cdh1Δ* cells were used for flow cytometry. (**D**) Asynchronous WT and *cdc20-1 FKH1-TAP* cells were treated with CHX for 1 hour. Proteins were harvested and Fkh1-TAP and Clb2 levels were determined. (**E**) Protein bands from three repeats of the experiment shown in (**D**) were quantified, normalized to - CHX samples and plotted. Standard error is shown.

The APC forms two complexes: a Cdc20-containing complex required for mitotic progression (APC^Cdc20^), and a Cdh1-containing complex that is required for G1 maintenance (APC^Cdh1^) [25, 42]. Cdc20 and Cdh1 are thought to recruit targets to the APC in order to be ubiquitinated [43]. To confirm that Fkh1 is specifically degraded during mitosis, we integrated Fkh1-TAP into the genomes of *cdh1*Δ and *cdc20-1* cells predicting that a *cdc20-1* mitotic mutant would disrupt Fkh1 recruitment and degradation in mitosis. *cdh1*Δ *FKH1-TAP* cells were chemically arrested in G1, treated with CHX, then assessed for Fkh1-TAP protein stability. Clb2 can be targeted by either APC^Cdc20^ or APC^Cdh1^ complex [44, 45], thus serves as a stability control as it should not be degraded at the specific cell cycle points defined by these mutants. We show that in *cdh1*Δ mutants, Fkh1-TAP levels remain low in G1, whereas Clb2 levels are markedly elevated, as predicted (Figs. 5A-C). This indicates that Fkh1-TAP is not targeted for degradation by the G1 specific APC^Cdh1-^since it remains low in *cdh1*Δ cells. On the other hand, in asynchronous *cdc20-1 FKH1-TAP* cells, both Clb2 and Fkh1-TAP proteins were clearly stabilized, in contrast to their degradation in WT cells (Figs. 5D, 5E). Lastly, we confirmed that the protein levels of Clb2 and Fkh1-TAP predictably rise and fall throughout the cell cycle following synchronous release of S phase-arrested cells back into the cell cycle. Our analysis confirmed that Fkh1-TAP levels began to drop soon after S phase arrest, prior to Clb2 levels, and began to accumulate before Clb2 (Fig. S2). Taken together, the above observations clearly demonstrate that Fkh1 turnover is cell cycle dependent, depends on the APC^Cdc20^ complex, and occurs at the onset of mitosis.

### Mutation of a highly conserved lysine residue stabilizes Fkh1

**Figure 6:**
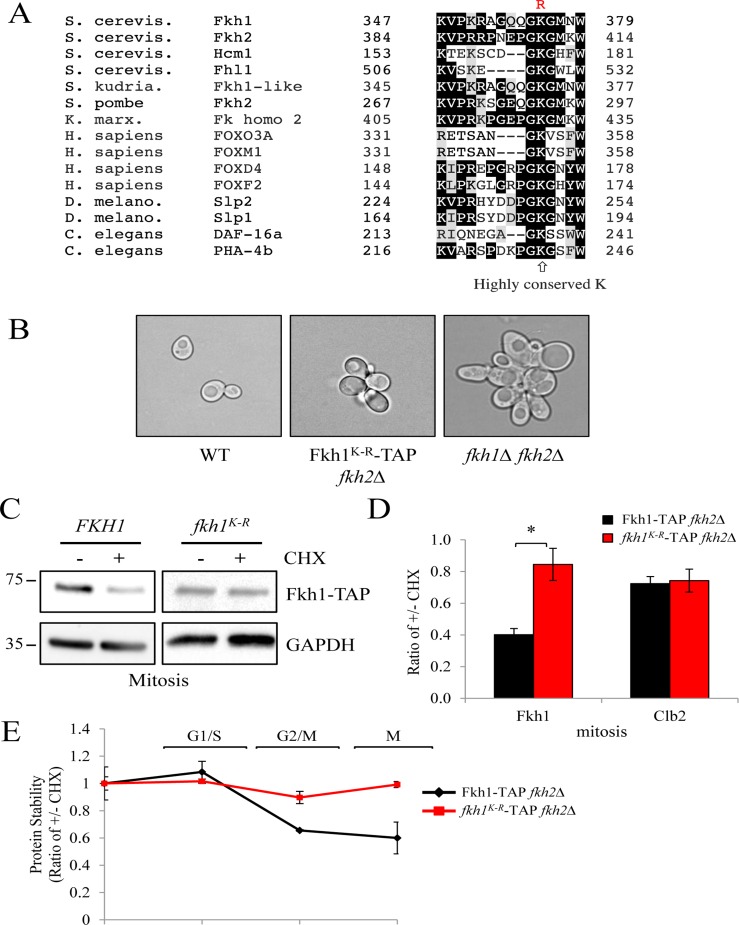
Mutation of the highly conserved K373 in Fkh1 stabilizes the protein in mitosis (**A**) Sequence alignment of Fkh1-like proteins across evolutionary boundaries highlighting the highly conserved lysine (K373). The mutation made is shown above in red. (**B**) Morphology of *fkh2Δ* cells harboring the *FKH1^K-R^* mutation shown in (**A**). (C) *fkh2Δ* cells expressing *FKH1^K-R^* were arrested in mitosis using nocodazole, then treated with CHX for 60 minutes. Proteins were then harvested and examined for Fkh1-TAP, Clb2 and GAPDH content. (**D**) All bands in (**C**) were imaged from three separate experiments and the ratio of intensities before and after CHX addition were determined. Standard error of the mean is shown. * - p>0.001. (**E**) *fkh2Δ* cells expressing *FKH1^K-R^* were arrested in S phase using HU, then released back into the cell cycle. Samples were removed every 30 minutes afterwards, treated +/− CHX for 1 hr, then proteins were recovered for Westerns. Bands from two experiments were scanned and the ratio of bands +/− CHX was plotted. See also Figure S3.

If Fkh1 is indeed targeted for degradation by the APC, then mutation of a ubiquitinated lysine (K) should abolish ubiquitination, and stabilize the protein. We searched Fkh1 for a conserved K, and found a highly conserved K at position 373 (Fig. 6A). We mutated the highly conserved K_373_ in the endogenously-TAP tagged *FKH1* gene to arginine (R) to generate the Fkh1^K-R^ strain. Since Fkh2 is believed to compensate for the loss of Fkh1 [37], we deleted *FKH2* in these cells to examine the isolated effects of the *FHK1* mutation. *fkh1^K-R^ fkh2*Δ cells were flocculent, but not as severely as *fkh1*Δ *fkh2*Δ cells (Fig. 6B), indicating that the K_373_R mutation impaired Fkh1 function, but did not completely disable it. Next, we tested whether the mutation altered Fkh1 mitotic protein instability. We observed that in cells arrested in mitosis, Fkh1 was unstable, whereas Fkh1^K-R^ was stable (Figs. 6C, 6D). Lastly, we determined the cycling profile of the Fkh1^K-R^ protein. The results show that the K_373_R mutation stabilized Fkh1 throughout the cell cycle (Figs. 6E, S3), indicating that K_373_ is required for mitotic Fkh1 instability.

**Figure 7:**
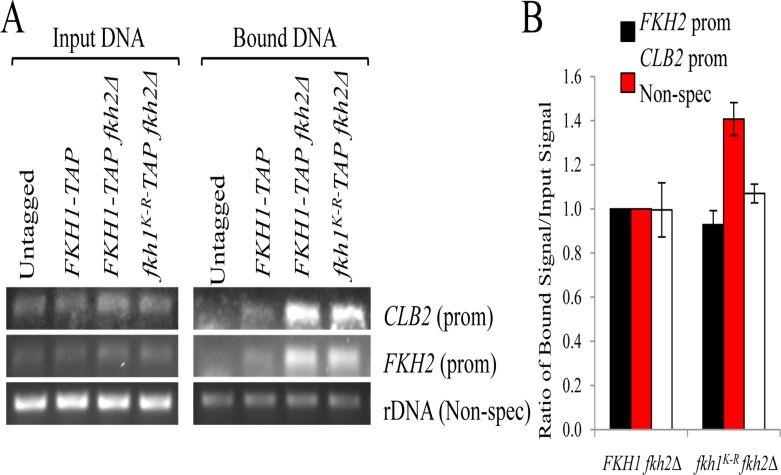
The Fkh1^K-R^ mutation does not impair recruitment of Fkh1 to *CLB2* and *FKH2* promoters (**A**) ChIP was performed on the strains shown. DNA was purified and used as template in PCR reactions using primers specific for the loci indicated. 36-38 cycles were determined to be in the linear range for these PCR reactions. DNA extracted prior to IP was used as input controls. (**B**) The DNA bands in (**A**) were scanned, quantified, and a ratio of bound:input DNA was plotted.

Given the flocculent phenotype of *fkh1^K-R^ fkh2*Δ cells (Fig. 6B), consistent with a defective Fkh1 protein, we asked if the K_373_R mutation impaired the core ability of Fkh1: binding promoter DNA. We used chromatin immunoprecipitation (ChIP) in asynchronous cells, with primers designed to amplify the *CLB2* and *FKH2* promoters, as Fkh1 binds these promoters (Fig. 7) [46]. The high abundance of rDNA, leading to nonspecific interactions with the beads, allows it to be used as a load control. In the bound lanes, Fkh1-TAP was observed at both *CLB2* and *FKH2* promoters, with Fkh1-TAP abundance clearly increased when *FKH2* was deleted (the *FKH2* promoter is still present in the *FKH2* coding region deletion). Thus, the ability of Fkh1 to compensate for Fkh2 [37] likely involves increased Fkh1 recruitment to promoters in the absence of *FKH2*. The stable Fkh1^K-R^ protein was bound to both *CLB2* and *FKH2* promoters at WT levels, demonstrating that the Fkh1^K-R^ defect, leading to flocculence in the *fkh2*Δ background, is not due to impaired promoter binding, at least at *CLB2* and *FKH2*.

### Fkh1 interacts physically with Apc5

**Figure 8:**
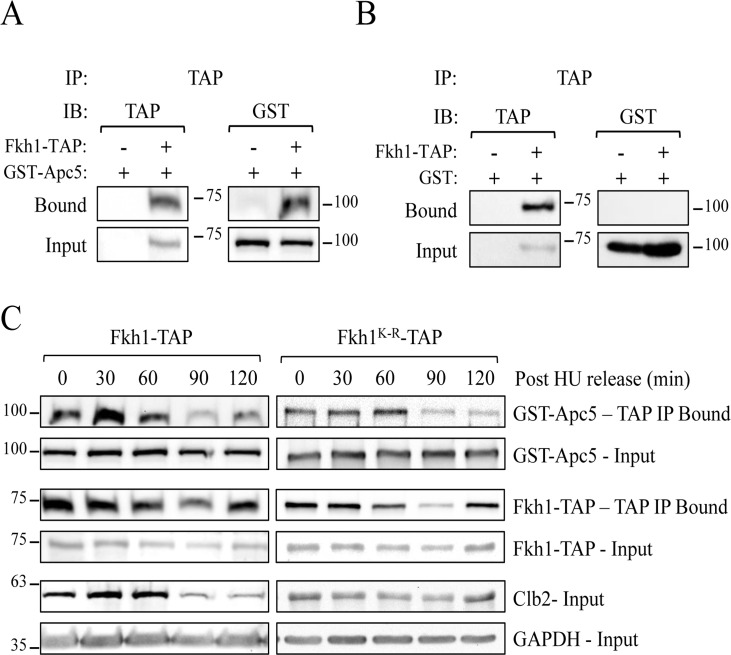
Fkh1 and Apc5 physically associate throughout the cell cycle (**A**) Fkh1-TAP cells expressing plasmid-borne *GST-APC5* were grown overnight to early log phase. Lysates were prepared and IPed using antibodies against the TAP epitope. Westerns using antibodies against TAP and GST were done. (**B**) Fkh1-TAP cells expressing the empty GST vector were used as described for (**A**). (**C**) The various Fkh1-TAP cells expressing plasmid-borne *GST-APC5* were arrested in S phase using HU. HU was then washed away, with cells released synchronously back into the cell cycle. Samples removed every 30 minutes were subjected to CoIP using TAP and Clb2 antibodies. Westerns were performed on input and bound samples using antibodies against TAP and GST. GAPDH was used as a load control. See also Figure S4.

If the APC targets Fkh1 for ubiquitination and degradation, then it is likely that Apc5 and Fkh1 physically interact. Cells expressing endogenous Fkh1-TAP and plasmid-borne GST-Apc5 were harvested after asynchronous log phase growth. Our results demonstrate that Fkh1 and Apc5 do in fact physically interact in asynchronous cells (Fig. 8A). The control using only GST shows that the TAP antibody does not interact with GST (Fig. 8B). Fkh1 protein levels do fluctuate predictably throughout the cell cycle, demonstrated by G1 arrest followed by synchronous release back into the cell cycle (Fig. S4A). In WT cells, Fkh1-TAP levels were lowest in G1, then steadily increased as the cell cycle progressed. These cell cycle-dependent fluctuations of Fkh1-TAP were blunted and minimized in APC mutants, as Fkh1 protein levels were higher in G1 arrested APC mutants (Figs. S4A, S4C).

We next asked if the Fkh1-Apc5 interaction is cell cycle dependent, perhaps preceding mitosis when Fkh1 is degraded. To determine this, we arrested the Fkh1-TAP cells in S phase, with samples removed for CoIP every 30 minutes after synchronous release back into the cell cycle (Fig. 8C). Apc5 co-immunoprecipitated with Fkh1 throughout the cell cycle, and its levels were proportional to Fkh1. Apc5 likewise continued to be observed in Fkh1^K-R^ precipitates, with levels also proportional to the amount of Fkh1 IP'd. Our observations therefore suggest that Fkh1 and Apc5 are physically bound throughout the cell cycle, even though Fkh1 degradation only occurs during mitosis, and the stabilizing K_373_R mutation is irrelevant for this interaction.

### Degradation of Fkh1 in mitosis is required for normal cell cycle progression through G1

**Figure 9:**
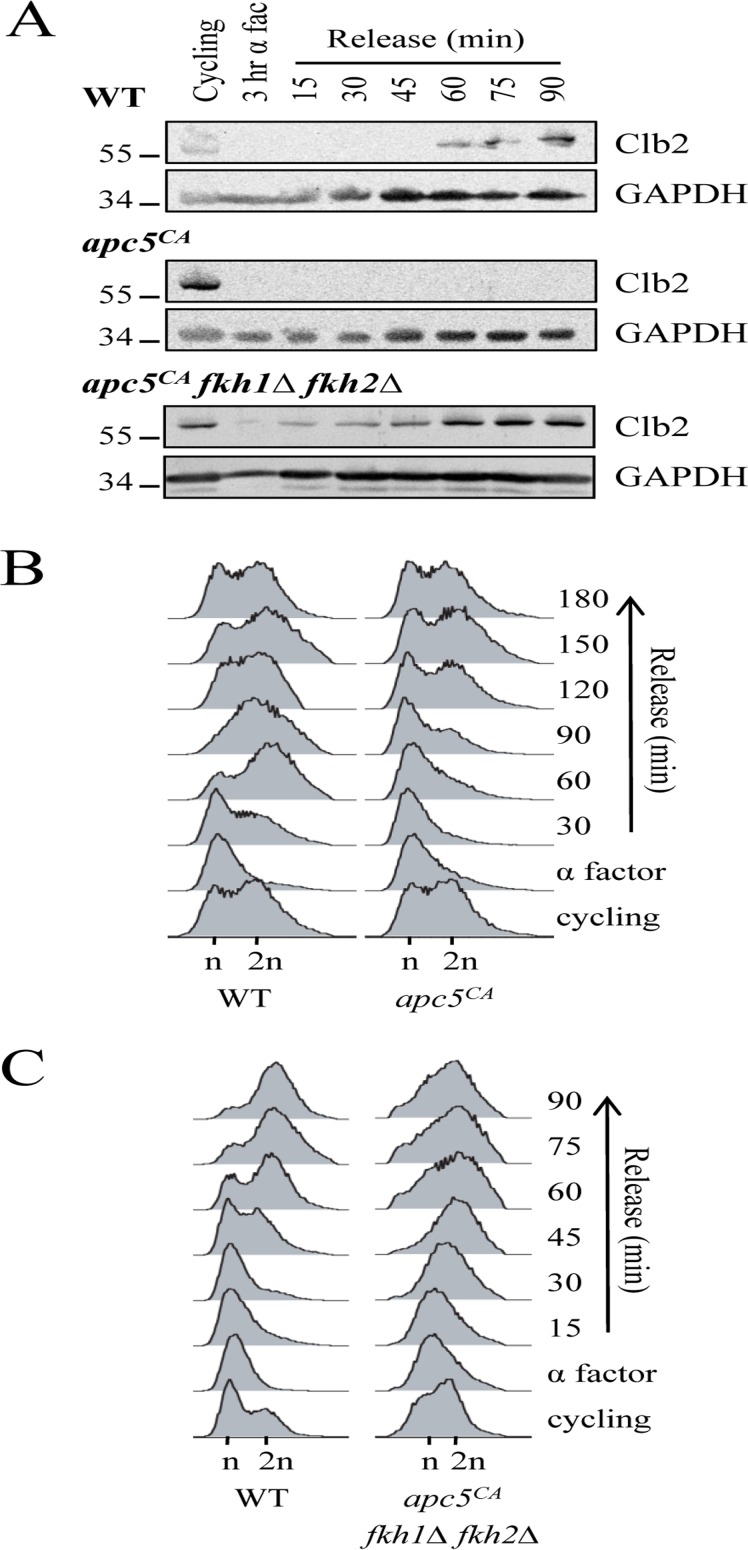
Removal of *FKH1* and *FKH2* re-establishes Clb2 synthesis and rapid cell cycle re-entry in *apc5^CA^* cells (**A**) WT, *apc5^CA^* and *apc5^CA^ fkh1Δ fkh2Δ* cells were arrested in G1 and then synchronously released back into the cell cycle. Samples were removed every 15 minutes for Western analyses using antibodies against Clb2 and GAPDH. (**B**) WT and *apc5^CA^* cells used for the experiment in (A) were allowed to grow for 3 hours with samples removed every 30 minutes for analysis by flow cytometry. (**C**) Flow cytometry was used on the WT and *apc5^CA^ fkh1Δ fkh2Δ* samples used in (A).

The demonstration that the APC is required to target Fkh1 for degradation leads to the next question: what is the biological significance of this interaction? We hypothesized that Fkh1 degradation in mitosis is necessary to facilitate ordered cell cycle progression into G1. Furthermore, we propose that this progression involves Fkh1-dependent transcriptional control of Clb2, based on a combination of several observations: asynchronous *apc5^CA^* mutants exhibit high Clb2 and Fkh1 protein levels (Figs. 2A, 2B) [6, 28]; G1 arrested *apc5^CA^* mutants exhibit very low Clb2 levels (Figs. 9A, S4A); and Fkh proteins normally repress *CLB2* expression during G1 [19, 23]. Together, this raises the possibility that the higher levels of Fkh1 protein (in G1 arrested APC mutants) could be keeping Clb2 protein levels low through transcriptional repression. To test this, WT, *apc5^CA^* and *apc5^CA^ fkh1*Δ *fkh2*Δ cells were arrested in G1, then released back into the cell cycle with samples removed to determine Clb2 protein levels (Fig. 9A). Our hypothesis predicts that removal of Fkh1/2 function in the mutant APC background will return Clb2 protein levels to near normal. In *apc5^CA^* mutants, Clb2 was not apparent until after 90 minutes, and it took 120 minutes for cells to begin to move into G2/M. However, in *apc5^CA^ fkh1*Δ *fkh2*Δ cells, Clb2 expression was clear after 15 minutes, with a synchronous movement of cells into S phase as early as 30 minutes (Figs. 9A, 9C). In WT cells, Clb2 protein levels begin to accumulate again after 60 minutes, which coincided with movement of cells from G1 to G2/M (Figs. 9A, 9B). These experiments, therefore, provide the biological relevance of Fkh1 degradation in mitosis as being essential for normal cell cycle progression.

### Stabilization of Fkh1 alters chronological lifespan and genomic stability

**Figure 10:**
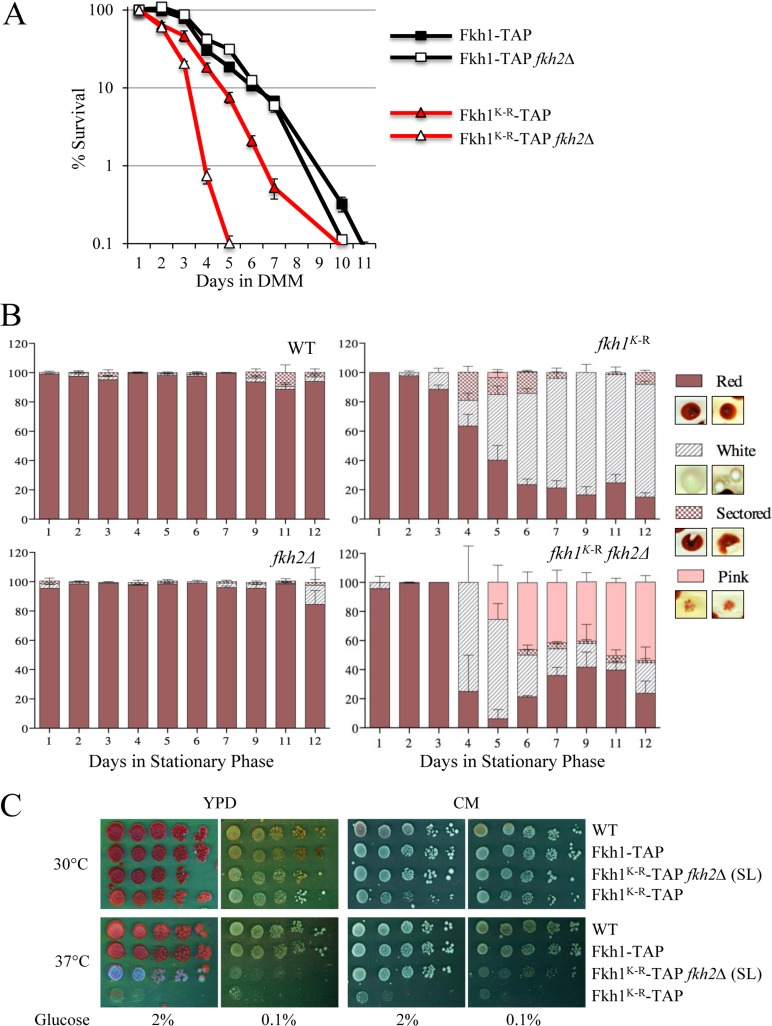
The Fkh1^K-R^ mutation decreases chronological lifespan, increases genomic instability, and confers temperature sensitive growth (**A**) Chronological lifespan (CLS) was performed on cells expressing the *FKH1^K-R^* allele, in the presence or absence of *FKH2*. Cells were grown in Minimal Media (MM) and maintained in the Depleted Minimal Media (DMM) throughout the experiment. The experiments were performed in duplicate, at least twice. Standard error of the mean is shown. (**B**) *FKH1^K-R^ ade2* cells, in the presence or absence of *FKH2*, and in stationary phase, reverted from red to white/sectored. Each day following stationary phase, cells were plated as part of the CLS experiment. The plates were left for a week to fully develop color. The number of cells that were white, sectored, or a mottled pink were counted and plotted as a percentage of the total. (**C**) Strains expressing *FKH1^K-R^*, +/− *FKH2*, were grown overnight in 2% YPD media, then spot diluted onto plates containing rich YPD or complete medium (CM). Plates were supplemented with either 2 or 0.1% glucose. The plates were incubated for 3 to 6 days at 30 and 37°C.

Additional important functions of the yeast Forkhead proteins includes lifespan and genomic stability, and here we assess whether Fkh1 protein stability is biologically important in these processes. Fkh1-TAP and Fkh1-TAP *fkh2*Δ cells exhibited near superimposable lifespans, when measured for chronological lifespan (CLS) (Fig. 10A). In contrast, *fkh1^K-R^ fkh2*Δ cells have a remarkably shorter lifespan, with 99% of the cell population dead by four days, compared to 99% dead by day nine in the *FKH1* WT strains. The presence of WT *FKH2* in *fkh1^K-R^* cells partially abrogated the shortening of the K-R lifespan, supporting its compensatory effect for Fkh1 function.

Color sectoring of yeast colonies is a marker of genomic instability, and we serendipitously observed that individual cells plated during the CLS experiment were sectoring, depending on genetic background. Colonies derived from the WT and *fkh2*Δ strains did not sector (Fig. 10B). Samples of sectored colonies are shown in the inset for Fig. 10B. However, the red *fkh1^K-R^ FKH2* mutant colonies generated significant numbers of sectored red:white, and white colonies. This occurs when the red phenotype of *ade2* mutants revert to white, due to spontaneous genomic mutation [47]. Mutations leading to red:white sectoring occur after plating, whereas the white colonies are due to mutations occurring prior to plating. The longer the cells sat in stationary phase, the more red:white colony sectoring occurred (Fig. 10B). Strikingly, it was most prevalent in the short-lived *fkh1^K-R^ fkh2*Δ cells. Pink speckled colonies began to appear suggesting that mutations causing color change were ongoing and persistent despite being in stationary phase. Thus, the lifespan defect in Fkh1^K-R^ cells appears to be linked with severe genomic instability.

Lastly, we questioned if stress response was impaired in the Fkh1^K-R^ mutant, along with its shortened lifespan and elevated genomic instability. We directly assessed this by spot diluting cells onto rich media (YPD) or complete media (CM) in the presence of temperature and carbon (2% vs 0.1% glucose) stress. Growth on 0.1% glucose requires metabolic adaptation to utilize alternative carbon sources; complete media (CM) is a much more nutritionally restricted growth medium than YPD; and higher temperatures contribute further to adverse growth conditions (Fig. 10C). In general we observed that *fkh1^K-R^ FKH2* cells were *ts* regardless of plate conditions. The shorter-lived *fkh1^K-R^ fkh2*Δ were more resilient, and were only *ts* when carbon stress was introduced (0.1% glucose). This investigation therefore confirms that Fkh1 degradation also plays an important biological role in carbon and temperature stress responses, which may be important for controlling genomic stability, as well as in the previous lifespan requirement. In conclusion, we have shown that Fkh1 is targeted for degradation by the APC^Cdc20^ complex in mitosis, and have identified a highly conserved lysine that when mutated results in reduced CLS, genomic instability and *ts* growth.

## DISCUSSION

In this report we establish that Fkh1 is an APC^Cdc20^ substrate targeted for degradation during mitosis. This is an important observation as it demonstrates that yeast Fox proteins are structurally and functionally conserved with higher eukaryotic systems. Considering the ease of yeast genetic manipulations, yeast will provide valuable insight into how Fox proteins are regulated at the molecular level. It is known that ubiquitination of Fox proteins plays a vital role in their regulation. It is primarily believed that nuclear Fox proteins are first phosphorylated, then shuttled from the nucleus to the cytoplasm, where they are targeted for degradation [48, 49]. Nuclear FOXO3A, for example, can be phosphorylated followed by Ub-dependent degradation by either AKT and the E3 SCF [50], or Erk and the E3 Mdm2 [51]. Likewise, FOXM1 protein is sequentially targeted by PLK1, then APC^Cdh1^ activity during G1 [52-54]. However, knowledge of the fine details of this regulation, and the result of defective regulation, are lacking. Here, we show that mutation of a highly conserved lysine residue that stabilizes Fkh1 also results in a shortened yeast lifespan, increased genomic instability, increased sensitivity to stress, and delayed progression through G1. Expression of the stabilizing *fkh1^K-R^* allele does not, however, alter Apc5/Fkh1 interactions, nor disrupt recruitment of Fkh1 to *CLB2* or *FKH2* promoters. These observations provide a framework for further investigations towards a thorough understanding of Fox protein function and regulation.

### Fkh1 is an APC substrate

Genetic interactions between *FKH* and APC mutants have been previously established, as deletion of both *FKH1* and *FKH2* exacerbates APC mutant cell cycle, lifespan and stress response phenotypes [5]. Nonetheless, while deletion of both *FKH1* and *FKH2* impair *apc5^CA^* defects, we show that mutation of only *FKH1* suppressed these defects (Fig. 1). Mutations that suppress APC defects have been proposed to define potential APC substrates, as removal of these proteins blocks their ability to accumulate and impair cell cycle function [32]. In addition, many proteins that activate the APC, later become APC substrates [24, 25]. Fkh1, therefore, constituted a likely APC substrate. Our previous observation that the Fkhs act upstream of the APC, as activators [5], provided further evidence to suggest that Fkh1 had the potential to be an APC substrate. Our results presented here provide compelling evidence to support this hypothesis, as Fkh1 **1)** accumu-lates and is stabilized in APC and proteasome mutants, and with proteasome inhibitors (Fig. 2), **2)** is reduced when APC subunits are overexpressed (Fig. 3), **3)** is unstable during mitosis (Fig. 4), **4)** is specifically stable in *cdc20-1*, but not *cdh1*Δ mutants (Fig. 5), and **5)** is stabilized when a highly conserved lysine is mutated to arginine (Fig. 6). The stabilized Fkh1^K-R^ protein resulted in reduced yeast CLS, increased genomic instability and increased sensitivity to stress (Fig. 10). Previous work from the Aparicio laboratory showed that Fkh1 and Fkh2 are required for early replication origin clustering, acting by facilitating long-range origin interactions [55]. More recently this lab showed that Fkh protein levels limit early origin firing, and that Fkh overexpression resulted in an increase in origin firing in early S phase. This increase was established in late G1 and slowed the progression of the replication fork [56]. Considering these observations, the inappropriate stabilization of Fkh1 during mitosis and G1 could lead to early firing of replication origins, thus further destabilizing cell cycle progression. Taken together, our results clearly show that the inability to target Fkh1 for degradation following mitotic onset has dire consequences to the cell.

Although Fox proteins are known in higher eukaryotic systems to be targeted for degradation, it was not at all clear in yeast how Fox proteins are regulated. Our detection of a physical interaction between Fkh1 with Apc5 lasting throughout the cell cycle supports the idea that Fkh1 is an APC substrate (Fig. 8). It is interesting that Apc5 and Fkh1 remain physically associated throughout the cell cycle, but that Fkh1 is only targeted for degradation during mitosis. One possible explanation for this association may be to allow the flexibility to rapidly respond to stress regardless of the cell cycle position. Both the APC and Fkh1 respond to stress [5, 57], thus it is possible a tight association of the two throughout the cell cycle allows for a rapid response to stress through the coordinated action of these two stress response pathways.

### Accumulation of Fkh1 during mitosis delays cell cycle re-entry

We have observed that Clb2 accumulates in asynchronous *apc5^CA^* cells, yet does not accumulate in G1 arrested *apc5^CA^* or *apc10Δ* cells (Figs. 2A, 4F, 9A, S4A). Knowing that Fkh1 can repress *CLB2* transcription in G1, we considered whether the low levels of Clb2 protein during G1 in APC mutants is a consequence of simultaneously elevated Fkh1 levels during G1. Our findings presented in Fig. 9 demonstrate that Clb2 protein synthesis and cell cycle progression through G1 was indeed delayed in *apc5^CA^* cells, supporting this concept. Interestingly, deletion of *FKH1* and *FKH2* in these cells not only reversed this effect, but accelerated Clb2 protein synthesis and cell cycle re-entry. Therefore, cell cycle progression through mitosis and G1 could rely on the downregulation of Fkh1.

In summary, our work presented here illuminates how Fkh1 function is regulated in a cell cycle-dependent manner. We identified a highly conserved Fkh1 lysine residue (K373) that regulates cell cycle-dependent protein stability, cellular lifespan, and genomic stability. This provides valued insight into how this family of conserved stress responsive transcription factors is regulated and what occurs when this regulation fails. Much remains to be learned regarding how the Fkhs interact in yeast with each other and with their binding partners. Our results therefore pave the way for future studies in higher eukaryotic systems.

## METHODS

### Yeast strains and plasmids

The *S. cerevisiae* strains used in this study are shown in Table 1. All strains were isogenic derivatives of S288c strains except where noted. BY4741 (YTH1029) and the *rpn10Δ::kanMX6* and *cdh1Δ::kanMX6* derivatives, were obtained from the ResGen Library (provided by W. Xiao, U of Saskatchewan). The *cdc20-1* strain and its associated WT (W303) were a kind gift from A. Amon (David Koch Institute for Integrative Cancer Research at MIT). PCR based methods were used to integrate the *TAP*::*HIS3* cassette into the various cells used. Cells harboring deletions marked by the same phenotypic marker were generated by crossing. The plasmid encoding *GAL*-driven *FKH1-HA* (hemaglutinin epitope tag) was obtained from Open Biosytems and kindly provided by W. Xiao. The copper-driven (50 μM CuSO_4_ for 2 hours) *GST-APC5*, *GST-APC10*, and the empty vector control (pYEX4T-1) were obtained from the Exclone Library.

**Table 1:** *Saccharomyces cerevisiae* strains used in this study

Strain	Genotype	Source
YTH457	*MATa ade2 his3 leu2 ura3 apc5CA-PA::His5*	Harkness *et al.* 2002
YTH1029	*MATa his3 leu2 met15 ura3*	W. Xiao
YTH1235	*MATa ade2 his3 leu2 lys2 ura3*	Harkness *et al.* 2002
YTH1693	*MATa his3 leu2 met15 ura3 apc10Δ::kanMX6*	Harkness *et al.* 2005
YTH2427	*MATa ade2 his3 leu2 lys2 met15 ura3 fkh1Δ::kanMX6*	Postnikoff *et al.* 2012
YTH2431	*MATa ade2 his3 leu2 lys2 met15 ura3 apc5^CA^-PA::His5 fkh1Δ::kanMX6*	Postnikoff *et al.* 2012
YTH2444	*MATa ade2 his3 leu2 lys2 met15 ura3 fkh2Δ::kanMX6*	Postnikoff *et al.* 2012
YTH2449	*MATa ade2 his3 leu2 lys2 met15 ura3 apc5^CA^-PA::His5 fkh2Δ::kanMX6*	Postnikoff *et al.* 2012
YTH2578	*MATa ade2 his3 leu2 lys2 met15 ura3 fkh1Δ::kanMX6 fkh2Δ::kanMX6*	Postnikoff *et al.* 2012
YTH2579	*MATα ade2 his3 leu2 lys2 met15 ura3 fkh1Δ::kanMX6 fkh2Δ::kanMX6*	Postnikoff *et al.* 2012
YTH2581	*MAT(?) ade2 his3 lys2 met15 ura3 apc5^CA^-PA::His5 fkh1Δ::kanMX6 fkh2Δ::kanMX6*	Postnikoff *et al.* 2012
YTH2582	*MATa ade2 his3 leu2 lys2 met15 ura3 apc5^CA^-PA::His5 fkh1Δ::kanMX6 fkh2Δ::kanMX6*	Postnikoff *et al.* 2012
YTH3124	*MAT(?) ade2 his3 leu2 lys2 met15 ura3 apc10Δ::kanMX6 fkh1Δ::kanMX6*	Postnikoff *et al.* 2012
YTH3346	*MAT(?) ade2 his3 leu2 lys2 met15 ura3 apc10Δ::kanMX6 fkh2Δ::kanMX6*	Postnikoff *et al.* 2012
YTH3408	*MAT(?) ade2 his3 leu2 lys2 met15 ura3 apc10Δ::kanMX6 fkh1Δ::kanMX6 fkh2Δ::kanMX6*	Postnikoff *et al.* 2012
YTH3409	*MAT(?) ade2 his3 leu2 lys2 met15 ura3 apc10Δ::kanMX6 fkh1Δ::kanMX6 fkh2Δ::kanMX6*	Postnikoff *et al.* 2012
YTH3638	*MATa his3 leu2 met15 ura3 rpn10Δ::kanMX6*	W. Xiao
YTH3785	*MATa his3Δ1 leu2 met15Δ ura3 cdh1Δ::kanMX6*	W. Xiao
YTH3862	*MATa ade2 ura3 leu trp1 his3 can1 GAL psi+*	A. Amon
YTH3863	*MATα ade2 ura3 leu2 trp1 his3 can1 cdc20-1*	A. Amon
YTH3926	as YTH1235, but *FKH1-TAP::HIS3*	Postnikoff *et al.* 2012
YTH3930	as YTH457, but *FKH1-TAP::HIS3*	Postnikoff *et al.* 2012
YTH3933	as YTH1693, but *FKH1-TAP::HIS3*	Postnikoff *et al.* 2012
YTH4004	as YTH457, but *bar1Δ::kanMX6*	Menzel *et al.* 2014
YTH4112	as YTH3785, but *FKH1-TAP::HIS3*	This study
YTH4117	as YTH1029, but *FKH1-TAP::HIS3*	This study
YTH4267	*MATa ade2 his3 leu2 ura3*	Menzel *et al.* 2014
YTH4373	as YTH3638, but *FKH1-TAP::HIS3*	This Study
YTH4379	as YTH3638, but *APC5-TAP::HIS3*	Islam *et al*. 2011
YTH4617	*MatA ade2 leu2 trp1 ura3 FKH1-TAP::HIS3*	This Study
YTH4675	as YTH1235, but *fkh1^K373R^TAP::HIS3*	This Study
YTH4753	*MatA ade2 lys2 met15 ura3 FKH1-TAP::HIS3 fkh2Δ::kanMX6*	This Study
YTH4755	*MatA ade2 lys2 met15 ura3 fkh1^K373R^TAP::HIS3 fkh2Δ::kanMX6*	This Study
YTH4798	as YTH4617, but *cdc20-1 Mat A*	This Study

### Media and methods

Yeast growth media used in this study, yeast/peptone/dextrose (YPD) and drop out (DO) supplemented with required amino acids, was described previously [5, 28]. Yeast Minimal Media (MM) used in chronological lifespan (CLS) experiments contained 0.17% yeast nitrogen base/0.5% ammonium sulfate/0.003% essential amino acids, and 2% glucose unless otherwise indicated, with the pH adjusted using sodium hydroxide. Yeast Complete Media (CM) was also used in CLS experiments (0.17% yeast nitrogen base/0.5% ammonium sulfate/2% glucose/0.13% dropout powder including all amino acids, and 1 sodium hydroxide tablet). Methods including spot dilutions, Westerns, reverse transcriptase PCR, *E. coli* and yeast transformations, flow cytometry, DNA mini-preps, oxidative stress plate assays, and replicative and chronological lifespan assays were all performed according to our previous publications [5, 28, 29, 58]. For MG132 experiments, cultures were started in YPD to ensure log phase growth, then transferred to synthetic media (0.17% yeast nitrogen base without ammonium sulfate) supplemented with 0.1% proline, essential amino acids, and 2% glucose, with addition of 0.003% SDS the next day for an additional 3 hours at 30°C. At this point 75 μM MG132 (Enzo Life Sciences) or the drug vehicle (dimethyl sulfoxide; DMSO) were added, and cells were incubated at 30°C for 3 hours before harvesting and storage at −80°C. Genomic instability was determined by scoring the number of red, white, red/white sectored and pink speckled colonies following stationary phase growth. Four separate plates were counted for each strain on each day. Antibodies against Clb2 (Santa Cruz Biotechnology), GAPDH (glyceraldehyde-3-phosphate dehydrogenase; Sigma), α-tubulin (Sigma), and Tandom Affinity Purification (TAP; Pierce) where purchased from the indicated suppliers. The GST J90 GST antibody was described previously [33].

### TAP Co-immunoprecipitation (Co-IP)

Lysates were generated using a standard beadbeat protocol [26] in RIPA buffer lacking SDS. TAP tagged proteins were IPed using IgG Sepharose 6 Fast Flow beads (GE Healthcare), essentially as described [33], and eluted lysates were analyzed by western blot to identify co-purified proteins. To generate lysates used in Co-IP, frozen cell pellets were resuspended in 2X cell volume of ice-cold RIPA (Radio Immuno Precipitation Assay Buffer) lysis buffer containing non-ionic detergents, a 1:500 dilution of protease inhibitor cocktail (BioVision), 10 μM n-ethylmaleimide, 1.2 mM dithiothreitol (DTT), and 5 mM EDTA. Iced 0.5 mm glass beads were added to the suspension and the frozen cells were mechanically disrupted using a Mini-Beadbeater (Biospec products). Lysates were clarified and total protein concentration was determined using the Bradford method (BioRad). Protein lysates were added to washed IgG Sepharose 6 Fast Flow beads (GE Healthcare) and rotated end-over-end at 4°C for 90 minutes. Following incubation, beads were washed 2X with lysis buffer, and proteins were eluted off beads by boiling in 2X Laemmli sample buffer and then SDS-PAGE was used to separate proteins for western analyses.

### Cell cycle arrest

Cell cycle arrest was performed according to our standard procedures [6, 21]. Hydroxyurea (Sigma) was used at 0.3 M for approximately 3 hours for S phase arrest. For mitotic arrest, Nocodazole (Calbiochem) was used for approximately 3 hours, with 5 μg/ml added initially and then an additional 2.5 μg/ml added after 90 minutes. For G1 arrest of *BAR1* strains, cells were first washed 2X with fresh YPD, and then resuspended in YPD at pH 3.5 containing 2 μg/ml of α-factor (Zymo Research) and cultured for approximately 3 hours, with an additional 2 μg/ml added after 90 min. Cultures were considered arrested once >90% of the cells were in the desired phase of the cell cycle according to bud morphology. To release cells from arrest, cultures were washed twice with fresh media. To stop protein synthesis Cycloheximide (CHX; Calbiochem) was used at 10 μg/ml. Arrests were confirmed by flow cytometry (Saskatoon Cancer Center/Pharmacy & Nutrition), as performed previously [5, 26].

### Quantitative PCR (Q-PCR)

Cells were cultured in YPD at 30°C, and samples were collected in mid log phase (ie.OD_600_=0.46-0.90). Equivalent cell amounts were harvested for each sample, for a total of 6×10^8^ total cells per sample. Total RNA was extracted using the hot phenol method with DNA digestion completed using DNAse I (Fermentas) in the presence of RNAse inhibitors (Fermentas). The quality of RNA was assessed spectrophotometrically by measuring the OD_260/280_ ratio. Only samples that measured a ratio between 1.8-2.0 were used. The RNA integrity was assessed by visual inspection of the 28S/18S subunits on a formaldehyde agarose gel. First-strand synthesis was performed on 1 μg of total RNA using RevertAid Reverse Transcriptase (Fermentas) following manufacturers’ recommendations. Gene-specific primers targeted against yeast *ACT1* and *FKH1* (Table 2) were designed based on full-length sequences available from the *Saccharomyces* Genome Database (SGD). The expression of *FKH1* was normalized with reference to the *ACT1* endogenous control gene. Standard PCR reactions were completed in the mid-linear range for each message using our previously published protocol [29].

**Table 2:** Primer pairs

Gene	Forward Primer	Reverse Primer
*FKH1*	5′-TCTCTTTCAGCAGTGGTG	5′-CTTGTACTCTTCCGGTAG
*ACT1*	5′-CGCCTCTGTGACAAGACAGC	5′-GTCGTAGTAGAAATTTGGCC
*FKH2* Promoter	5′-CACTTCAAAGCCAATCTTCCC	5′-TTCTTGCGCATTGATAAAGT
*CLB2* Promoter	5′-GACAGATTTTATTCCAAATGCGG	5′-CGCTTTTCAGAAGTATCAATTCG
*FKH1^K373R^*	5′-GTGCCCAAAAGAGCCGGCCAACAAGGTAGAGGG	5′-TTTCCAATTCATCCCTCTACCTTGTTGGCCGGCTC
*FKH1^DB^*	5′-TGGCAGAACTCCGTTGCGCATAATGCGTCC	5′-GGCTTTATTTAGGGACGCATTATGCGCAACGG
rDNA Internal	5′-TAAGCGGAGGAAAAGAAACC	5′-CTCTTCGAAGGCACTTTACA

### Chromatin Immunoprecipitation (ChIP)

CHIP was performed with modifications to those previously described [59]. Following cross-linking of the cell lysate (1% formaldehyde at room temperature for 15 min), and quenching of the cross-linker (5 min incubation in 125 mM glycine) cells were washed 2× with iced TBS (20 mM Tris HCL pH 7.6, 150 mM NaCl) and frozen at −80°C. Frozen cells were resuspended in lysis buffer (50 mM Hepes-KOH pH 7.6, 150 mM NaCl, 1 mM EDTA, 1% Triton X-100, 0.1% Na deoxycholate, 1:500 dilution of protease inhibitor cocktail (BioVision)) and lysed by mechanical disruption using iced 0.5 mm glass beads with two 30-sec pulses in a Mini-BeadBeater (Biospec Products). Lysates were sonicated 5 × 10 sec at output 2 (Branson Sonifier 250). Sonicated lysates were clarified and total protein was quantified using the Bradford method (BioRad). Equivalent amounts of total protein were incubated with washed IgG Sepharose 6 Fast Flow beads (GE Healthcare) and rotated end-over-end at 4°C for 90 minutes. The IPs were washed 2× with lysis buffer, 1x with lysis buffer with 500 mM NaCl, 1x with CHIP wash buffer (10 mM Tris-HCl pH 8.0, 0.25 M LiCl, 0.5% NP-40, 0.5% Na deoxycholate, and 1 mM EDTA), and 1x with TE (10 mM Tris-HCl pH 8.0, 1 mM EDTA). Samples were eluted from the beads by incubating for 15 min at 65°C with 50 mM Tris-HCl pH 8.0, 10 mM EDTA, and 1% SDS. The beads were then washed with TE/0.67% SDS and the wash was pooled with the eluted sample. Cross-links were reversed by overnight incubation at 65°C, followed by protein digestion with Proteinase K (Sigma), and RNA digestion with RNAse A (Sigma). DNA was phenol-extracted, ethanol-precipitated, and resuspended in H_2_O. Crude input samples were made up to 250 μL with TE/1% SDS and the cross-links were reversed and the DNA purified as indicated above.

Equivalent amounts of input DNA for each sample were used in PCR. Two sets of primers were used in each reaction. Primer sets were designed to amplify the promoter region of *CLB2* or *FKH2*, or part of the *RDN1* coding sequence, which served as a loading control. PCR involved an initial denaturation of 30 sec at 98°C, then 36-40 cycles at: 98°C for 15 sec, 55°C for 20 sec, and 72°C for 20 sec, with a final extension at 72°C for 5 min. Control PCR reactions were carried out with a range of cycles to ensure that we were in the linear portion of the assay.

### Statistics

The Standard Error of the Mean was calculated in Microsoft Excel and was used for error bars. Significance was calculated using the Student T-test for comparing 2 Independent Means (http://www.socscistatistics.com).

## SUPPLEMENTAL FIGURES


