# Supplementary material for: Mitotic degradation of yeast Fkh1 by the Anaphase Promoting Complex is required for normal longevity, genomic stability and stress resistance
Source: Aging (Albany NY). 2016 Apr 19;8(4):810–30. doi: 10.18632/aging.100949 (PMC4925830; doi:10.18632/aging.100949)
Supplement: Supplementary file 1 [file aging-08-810-s001.pdf]

SUPPLEMENTAL FIGURES

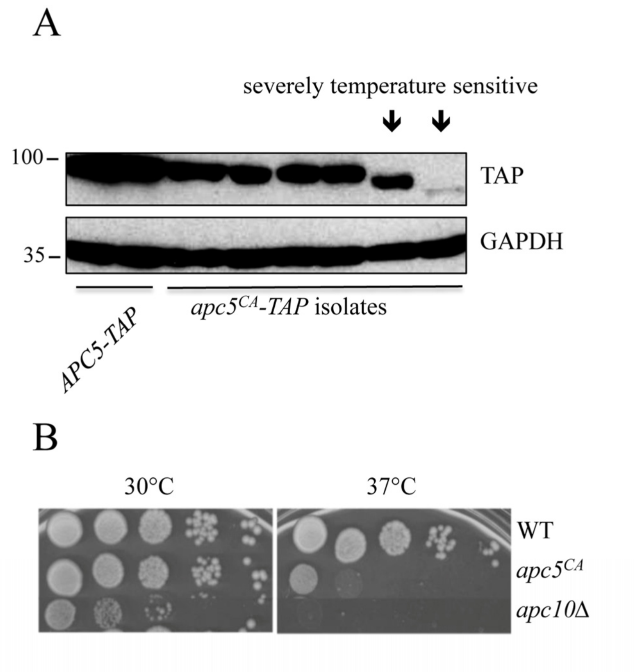

Supplemental Figure 1. APC mutant characteristics. (**A**) The TAP epitope was integrated at the C-terminus of the endogenous *APC5* and *apc5*^{CA} alleles using a PCR-based homologous recombination strategy. *apc5*^{CA}*-TAP* isolates with different sensitivities to growth at 37°C were selected and used for Western analyses with antibodies against TAP and GAPDH. (**B**) *apc5*^{CA} and *apc10Δ* mutants were spot diluted onto YPD plates with growth compared at 30°C and 37°C.

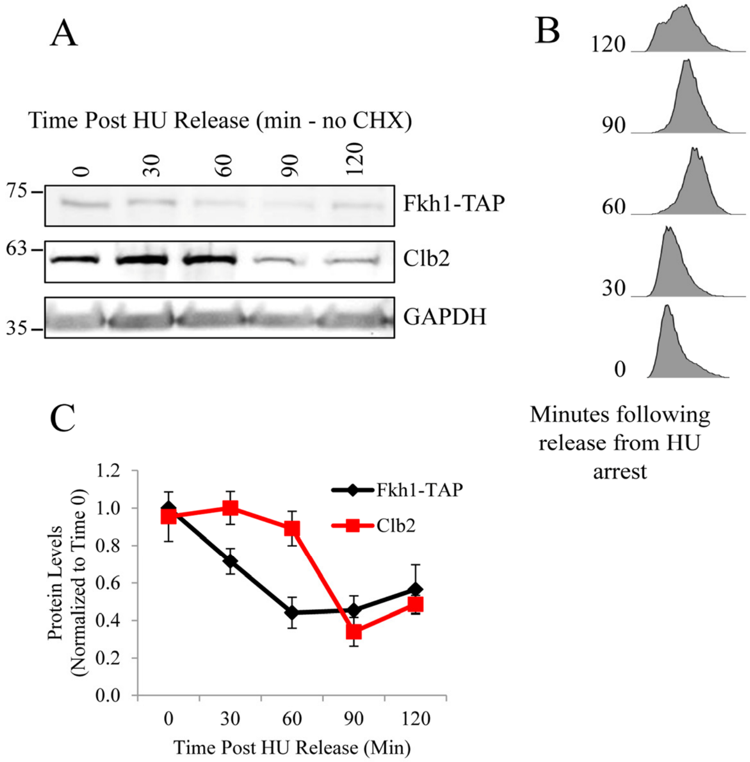

Supplemental Figure 2. Fkh1 instability occurs prior to Clb2 instability. (**A**) Fkh1-TAP expressing cells were arrested in S phase using hydroxyurea, washed and synchronously released back into the cell cycle in fresh media. Samples were removed every 30 minutes, with Fkh1 and Clb2 levels assessed using Western blotting. (**B**) Samples from (**A**) were also used for flow cytometry. (**C**) Protein bands were from (**A**) were quantified, normalized to time 0 and plotted. The experiment was repeated 3 times, with standard error shown.

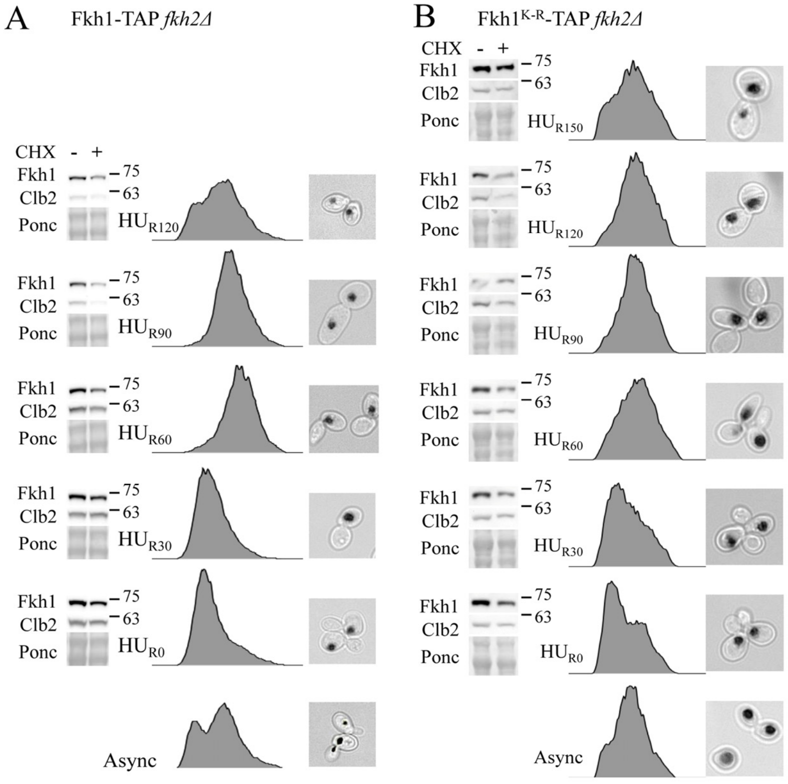

Supplementary Figure 3. The Fkh1^{K-R} mutation stabilizes the protein in mitosis. See also Fig. 6E. (**A**) *fkh2Δ* cells expressing the wild type version of Fkh1-TAP were arrested in S phase using hydroxyurea. Cells were synchronously released back into the cell cycle with samples removed every 30 minutes for Western analyses and flow cytometry. Merged images of cells stained with propidium iodide were included to show typical cell morphology at each time point. The data in this figure was used to generate the graph in Fig. 6E. (**B**) The experiment performed in (**A**) was repeated using Fkh1^{K-R}-TAP *fkh2Δ* cells.

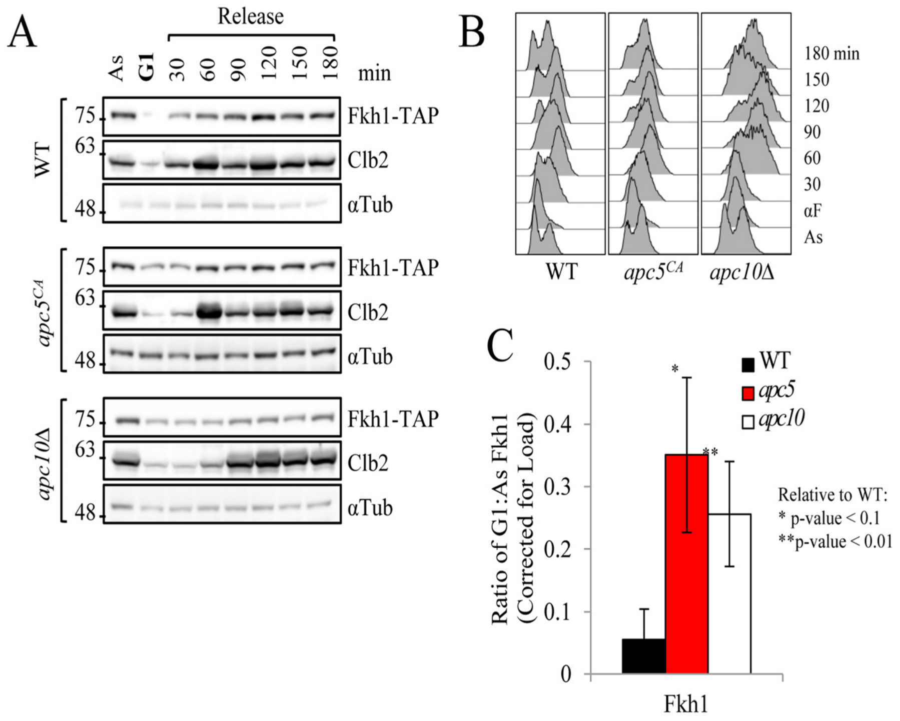

Supplementary Figure 4. Fkh1 levels fluctuate throughout the cell cycle, with levels lowest during G1. See also Fig. 8. (**A**) Wild type, *apc5^{CA}* and *apc10Δ* cells expressing *FKH1-TAP* were grown to mid log, then arrested in G1 using α-factor. The cells were then allowed to synchronously re-enter the cell cycle. Samples were removed every 30 minutes for 3 hours for Westerns. (**B**) Samples from the experiment shown in (A) were removed for flow cytometry. (**C**) The bands corresponding to the asynchronous (As) and G1 arrested lanes in (**A**) were scanned, quantified, normalized to load, then presented as a G1:As ratio. At least three separate experiments were performed, with standard error of the mean presented.
